# Amylin and beta amyloid proteins interact to form amorphous heterocomplexes with enhanced toxicity in neuronal cells

**DOI:** 10.1038/s41598-020-66602-9

**Published:** 2020-06-25

**Authors:** Prashant Bharadwaj, Tanya Solomon, Bikash R. Sahoo, Katarzyna Ignasiak, Scott Gaskin, Joanne Rowles, Giuseppe Verdile, Mark J. Howard, Charles S. Bond, Ayyalusamy Ramamoorthy, Ralph N. Martins, Philip Newsholme

**Affiliations:** 1 School of Pharmacy and Biomedical Sciences, Curtin Health and Innovation Research Institute (CHIRI), Faculty of Health Sciences, Curtin University, Bentley, WA 6107 Australia; 2 Centre of Excellence for Alzheimer’s disease Research and Care, School of Medical and Health Sciences, Edith Cowan University, Joondalup, WA 6027 Australia; 3 Biophysics and Department of Chemistry, Biomedical Engineering, Macromolecular Science and Engineering, University of Michigan, Ann Arbor, MI 48109-1055 USA; 4 School of Molecular Sciences, The University of Western Australia, Crawley, WA 6009 Australia; 5 Centre for Microscopy, Characterisation and Analysis, The University of Western Australia, Crawley, WA 6009 Australia; 6 School of Chemistry, University of Leeds, Leeds, LS2 9JT UK; 7 School of Biomedical Science, Macquarie University, Sydney, NSW Australia

**Keywords:** Alzheimer's disease, Neurodegeneration

## Abstract

Human pancreatic islet amyloid polypeptide (hIAPP) and beta amyloid (Aβ) can accumulate in Type 2 diabetes (T2D) and Alzheimer’s disease (AD) brains and evidence suggests that interaction between the two amyloidogenic proteins can lead to the formation of heterocomplex aggregates. However, the structure and consequences of the formation of these complexes remains to be determined. The main objective of this study was to characterise the different types and morphology of Aβ-hIAPP heterocomplexes and determine if formation of such complexes exacerbate neurotoxicity. We demonstrate that hIAPP promotes Aβ oligomerization and formation of small oligomer and large aggregate heterocomplexes. Co-oligomerized Aβ42-hIAPP mixtures displayed distinct amorphous structures and a 3-fold increase in neuronal cell death as compared to Aβ and hIAPP alone. However, in contrast to hIAPP, non-amyloidogenic rat amylin (rIAPP) reduced oligomer Aβ-mediated neuronal cell death. rIAPP exhibited reductions in Aβ induced neuronal cell death that was independent of its ability to interact with Aβ and form heterocomplexes; suggesting mediation by other pathways. Our findings reveal distinct effects of IAPP peptides in modulating Aβ aggregation and toxicity and provide new insight into the potential pathogenic effects of Aβ-IAPP hetero-oligomerization and development of IAPP based therapies for AD and T2D.

## Introduction

Epidemiological and clinical evidence suggests a strong association between Type 2 diabetes (T2D) and Alzheimer’s disease (AD), with T2D patients having a significantly higher risk of developing AD compared to non-diabetic individuals^1–3^. Both AD and T2D share common pathogenic markers including inflammation, oxidative stress, metabolic dysfunction and accumulation of the amyloidogenic proteins including beta amyloid (Aβ) and pancreatic islet amyloid polypeptide (IAPP or amylin)^4,5^. Aβ is the main component of senile plaques in the AD brain^6^. Similar to Aβ, human IAPP (hIAPP) containing deposits are observed in the T2D pancreas^7^. The accumulation of aggregated Aβ or hIAPP in the brain and pancreas has been shown to be associated with cell dysfunction and death^8,9^. Aβ is produced via the N-terminal proteolytic cleavage of amyloid precursor protein (APP) by BACE1 and at the C-terminus by γ-secretase, with the final Aβ length varying from 39 to 43 amino acids^10^. Aβ42 is the central component of senile plaques observed in the AD brain and is considered the main neurotoxic form^11,12^. hIAPP is cleaved from the pre-pro form of IAPP and subsequently converted into a 37-amino acid structure by pancreatic β-cell secretory granules^7^. Mature hIAPP modulates insulin-sensitive glucose uptake and glucose metabolism within peripheral tissues, as well as playing a role in the brain in processes such as satiety signalling and gastric emptying^3,13,14^.

Growing evidence suggests that cross-seeding interactions of Aβ and IAPP in the brain as one of the main pathways underlying the risk of neurodegeneration and AD in T2D. Multiple studies have identified hIAPP in both diabetic and dementia patients^15–17^ and the Fawver *et al*. study in particular, identified hIAPP in the cerebrospinal fluid (CSF) and brains of AD and T2D patients^16^. In addition, co-localised hIAPP and Aβ deposits have been observed in post-mortem human brains^16^. hIAPP has also been identified in the temporal lobe grey matter in diabetic patients and IAPP/Aβ deposits were occasionally observed to be co-localised^15^. Consistent with the co-localisation of hIAPP and Aβ within the brain, *in vitro* studies have demonstrated that hIAPP interacts with Aβ, functioning as a seed to promote aggregation, indicating its potential pathogenic role in promoting Aβ cross-seeding and amyloid deposition in AD and T2D^18^.

Despite a lack of association between their biological functions, hIAPP and Aβ share a sequence similarity of 50%, identity of 25% and with the highest similarity located within the β-sheet regions associated with fibril formation^19,20^. Structural studies involving molecular dynamic simulations of pre-existing Aβ and IAPP fibril models have predicted the interactions between Aβ and hIAPP and demonstrated that these oligomers have the potential to form heterocomplex structures^21–23^. From these models, it is proposed that IAPP and Aβ tetramers, pentamers and hexamers interact to form octamer, decamer and dodecamer heterocomplexes^21,24,25^. However, the size distribution, morphology of the Aβ-IAPP heterocomplexes, and if they have modified toxicity in neuronal cells, is largely undetermined^26,27^.

The overall aim of this study was to investigate the size distribution, morphology and more importantly the neurotoxic effects of the co-oligomerized Aβ-hIAPP heterocomplexes, in comparison to Aβ42 and hIAPP. We hypothesize that hIAPP promotes Aβ oligomerization and formation of distinct heterocomplex aggregates with increased ability to promote cell death in neurons, as compared to Aβ alone.

## Results

### hIAPP promotes Aβ42 oligomerization and formation of large aggregates

**Figure 1:**
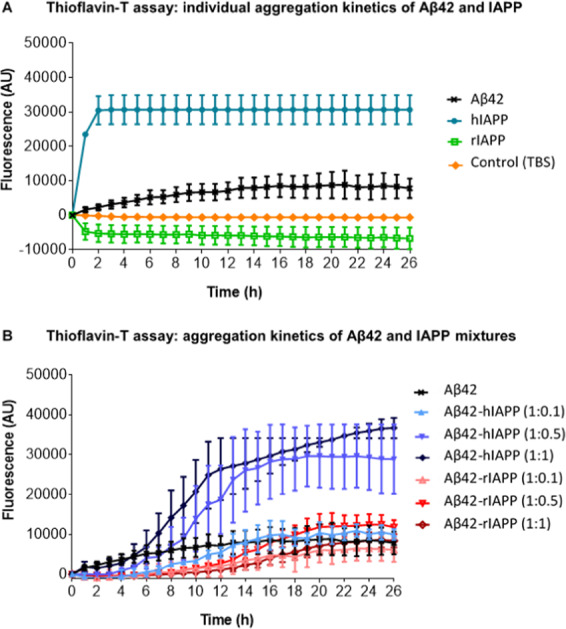
Aggregation kinetics of Aβ42-IAPP mixtures. (**A**) Individual aggregation kinetics of disaggregated Aβ42, hIAPP and rIAPP (20 µM) alongside TBS control assessed by Thioflavin-T (ThT) fluorescence over a 26 h period (mean ± SEM, n = 3, p < 0.001). (**B**) Aggregation kinetics of Aβ42 (20 µM) co-incubated with different concentrations (1:0.1, 1:0.5, 1:1) of hIAPP and rIAPP assessed by ThT fluorescence over 26 h. ThT fluorescence is represented as arbitrary units (AU; mean ± SEM, n = 3, p < 0.001). With increasing concentrations of hIAPP, Aβ42-hIAPP mixtures demonstrate a dose-dependent increase in ThT fluorescence. Aβ42-rIAPP mixtures demonstrate low ThT fluorescence at all concentrations compared to Aβ42, but this effect was not significant.

hIAPP is highly amyloidogenic and can act as a seed for Aβ aggregation^18^. It is likely that Aβ and hIAPP cross-seeding would be dependent on their respective structural similarities and intermolecular interactions, but the underlying molecular mechanisms are poorly understood at present. To investigate cross-seeding interactions of Aβ42 and hIAPP, we initially assessed the aggregation of Aβ42, hIAPP and a non-amyloidogenic IAPP control (rat amylin, rIAPP) individually and in combinations using Thioflavin-T (ThT) assays (Fig. 1).

We first determined the aggregation profiles of freshly prepared Aβ42, hIAPP or rIAPP peptides (non-oligomerized) incubated with ThT at 0 and every 2 h thereafter up to 26 h (Fig. 1A). Values from 0 h to 26 h were normalized by subtracting the baseline value (t = 0) from all values to represent relative increase in fluorescence. Compared to Aβ42, and rIAPP, hIAPP aggregated very rapidly. A steep increase in hIAPP aggregation was observed from 2 h which then plateaued after 4 h. Whereas, a more gradual and modest increase in aggregation that plateaued following 12 h incubation period was observed for Aβ42. This is consistent with the higher propensity of hIAPP to aggregate than Aβ42^28^. In contrast to Aβ42 and hIAPP, no increase in aggregation was observed with rIAPP, consistent with its markedly lower propensity to form aggregates^29^.

We next determined if hIAPP altered the time-course aggregation profile of Aβ42 (Fig. 1B). Aβ42 was co-incubated with increasing concentrations of hIAPP [Aβ42: hIAPP ratios of 1:0.1, 1:0.5 or 1:1]. The aggregation profile was compared to that observed for Aβ42 co-incubated with rIAPP at the same ratios, or to Aβ42 in the absence of IAPP peptides. Co-incubating Aβ42 with hIAPP at ratio of 1:05 led to a marked increase in Aβ42 aggregation, particularly seen at the 10 h time point. Further increases in Aβ42 aggregation was observed at equimolar ratio of Aβ42: hIAPP. Comparing Aβ42 and Aβ42: hIAPP aggregation profile (Fig. 1A,B), it could also be concluded that Aβ42 decreased the rate of hIAPP aggregation, particularly at equimolar ratios. Unlike hIAPP, no increase in aggregation was observed for Aβ42 co-incubated with rIAPP. In fact, at equimolar Aβ42: rIAPP ratio, a trend towards reduced aggregation compared to Aβ42 alone was observed (Fig. 1B). Overall, findings from ThT fluorescence assays demonstrated that hIAPP increased Aβ42 aggregation, whereas rIAPP trended towards slightly reducing it. Overall these suggests that both IAPP peptides interact with Aβ42 modulating its aggregation into distinct folding pathways.

**Figure 2:**
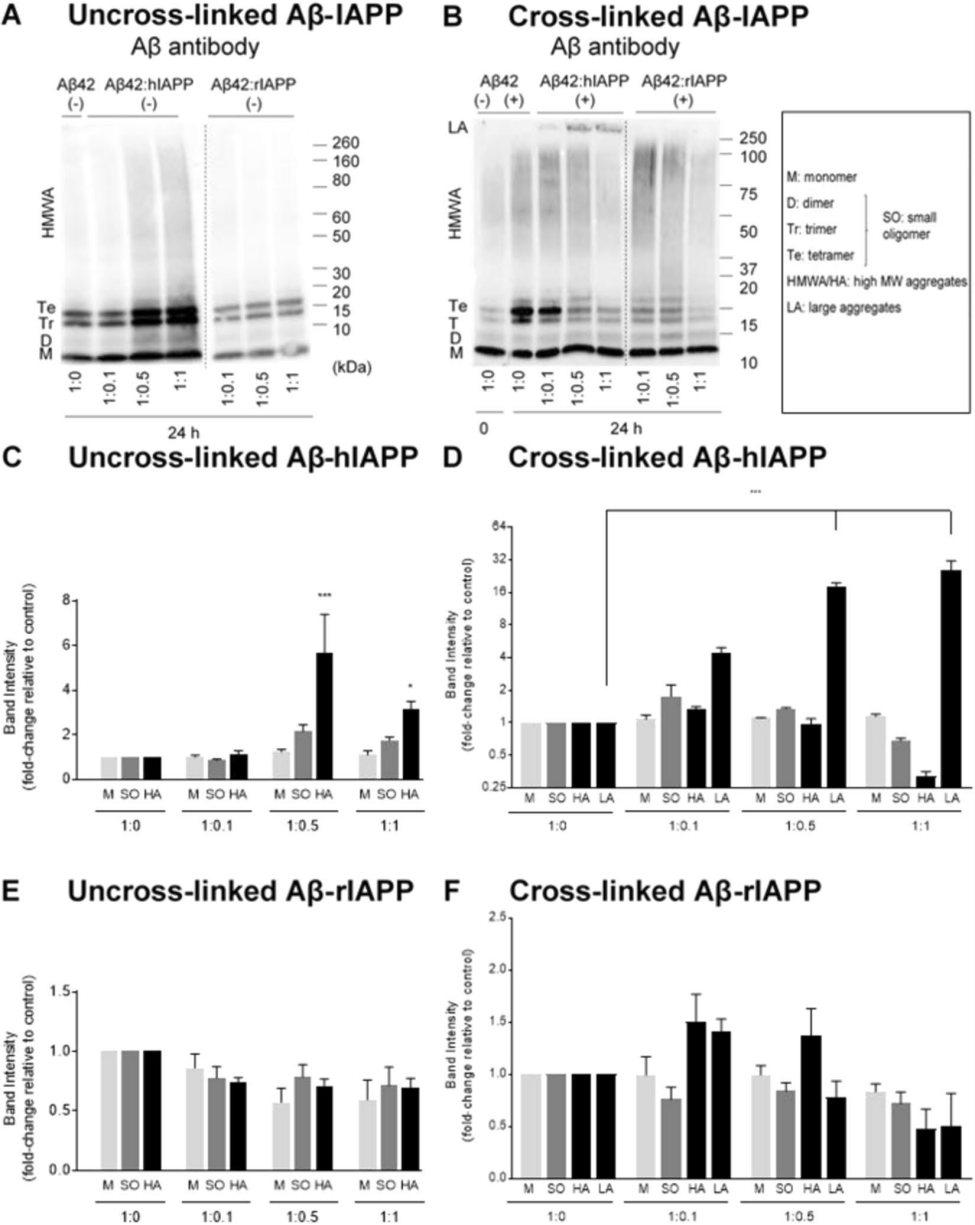
Oligomerization and size distribution of uncross-linked and photo cross-linked Aβ42-IAPP mixtures. Western immunoblotting analysis of (**A**) uncross-linked (−) and (**B**) cross-linked (+) Aβ42-IAPP at 24 h time-point assessed using Aβ antibody WO2. Densiometric analysis of (**C**) Aβ42-hIAPP (−), (**D**) Aβ42-hIAPP (+) (**E**) Aβ42-rIAPP (−) and (**F**) Aβ42-rIAPP (+) mixtures at 24 h timepoint to determine levels of monomers (4.5 kDa), small oligomers (9–17 kDa), HMW aggregates (40–160 kDa) and large aggregates (>250 kDa). Marked increase in the formation of small oligomers (2-fold) and HMW aggregates (6-fold) were observed in Aβ42:hIAPP mixtures of 1:0.5 and 1:1 ratios, while Aβ42-rIAPP mixtures did not demonstrate any significant changes as compared to Aβ42 alone. Band intensities represented as fold-change relative to control (mean ± SEM (n = 5), *p < 0.005, ***p < 0.001). Cross-linked samples of Aβ42 incubated with higher concentrations of hIAPP (1:0.5, 1:1) demonstrated a significant 20-fold increase in large aggregates (>250 kDa), combined with a decrease in the levels of oligomers (**B,D**). An increase in HMW aggregates in Aβ42:rIAPP (1:0.1) and to a lesser extent Aβ42:rIAPP (1:05) was observed, but was not significant (**B**). Dotted lines indicate spliced bands from the same gel in which blank lanes were removed.

To confirm the changes observed in Aβ42 aggregation profile observed with hIAPP or rIAPP, denaturing SDS-PAGE followed by immunoblotting was used to detect the formation of oligomers. Samples from co-oligomerized Aβ42-IAPP mixtures were analysed by SDS-PAGE and western immunoblotting using antibodies against Aβ (WO2) (Fig. 2). The levels of monomer (4.5 kDa), small oligomers (dimer 9 kDa, trimer 13.5 kDa and tetramer 17 kDa) and higher molecular weight (HMW) aggregates (40–160 kDa) were quantified in the Aβ42-IAPP mixtures and compared to those observed in samples of Aβ42 only (Fig. 2A,C,E). Marked increases in the formation of small oligomers (2-fold) and HMW aggregates (6-fold) were observed in Aβ42: hIAPP mixtures of 1:0.5 and 1:1 ratio (Fig. 2A,C), while Aβ42-rIAPP mixtures did not demonstrate any significant changes as compared to Aβ42 alone (Fig. 2A,E). In addition, a decreasing non-significant trend in the levels of monomer, small and HMW aggregates was observed in Aβ42-rIAPP at all concentrations as compared to Aβ42 only (Fig. 2A,E).

We also used photo-induced cross-linking (PICUP) to assess whether Aβ42-hIAPP mixtures formed any metastable, intermediate aggregates that may be undetectable without cross-linking. PICUP has been previously utilised to covalently cross-link and stabilise metastable protein complexes that are prone to disaggregation by SDS and heat denaturing treatments^30^. Aβ42-IAPP mixtures were cross-linked using PICUP, followed by SDS-PAGE and western immunoblotting analysis. As expected, increased levels of oligomeric species were observed in cross-linked (CL) Aβ42 samples compared to uncross-linked (un-CL) samples (Fig. 2B,D,F). CL Aβ42 demonstrated increased levels of small and HMW aggregates as compared to un-CL Aβ42 (compare Fig. 2A,B). Cross-linked samples of Aβ42 incubated with higher concentrations of hIAPP (1:0.5, 1:1) demonstrated a significant 20-fold increase in large aggregates (>250 kDa), combined with a decrease in the levels of oligomers (Fig. 2B,D). It is also notable that these large aggregates observed in CL Aβ42-hIAPP mixtures (Fig. 2B) were absent in un-CL Aβ42-hIAPP samples (Fig. 2A) suggesting that these large aggregates were intermediate Aβ42-hIAPP complexes probably disaggregated by SDS and heat denaturation and therefore undetectable in the un-CL samples. An increase in HMW aggregates in Aβ42:rIAPP (1:0.1) and to a lesser extent Aβ42:rIAPP (1:05) was observed, but was not significant (p > 0.05) (Fig. 2B).

### Aβ42 and hIAPP interact to form distinct heterocomplex aggregates

**Figure 3:**
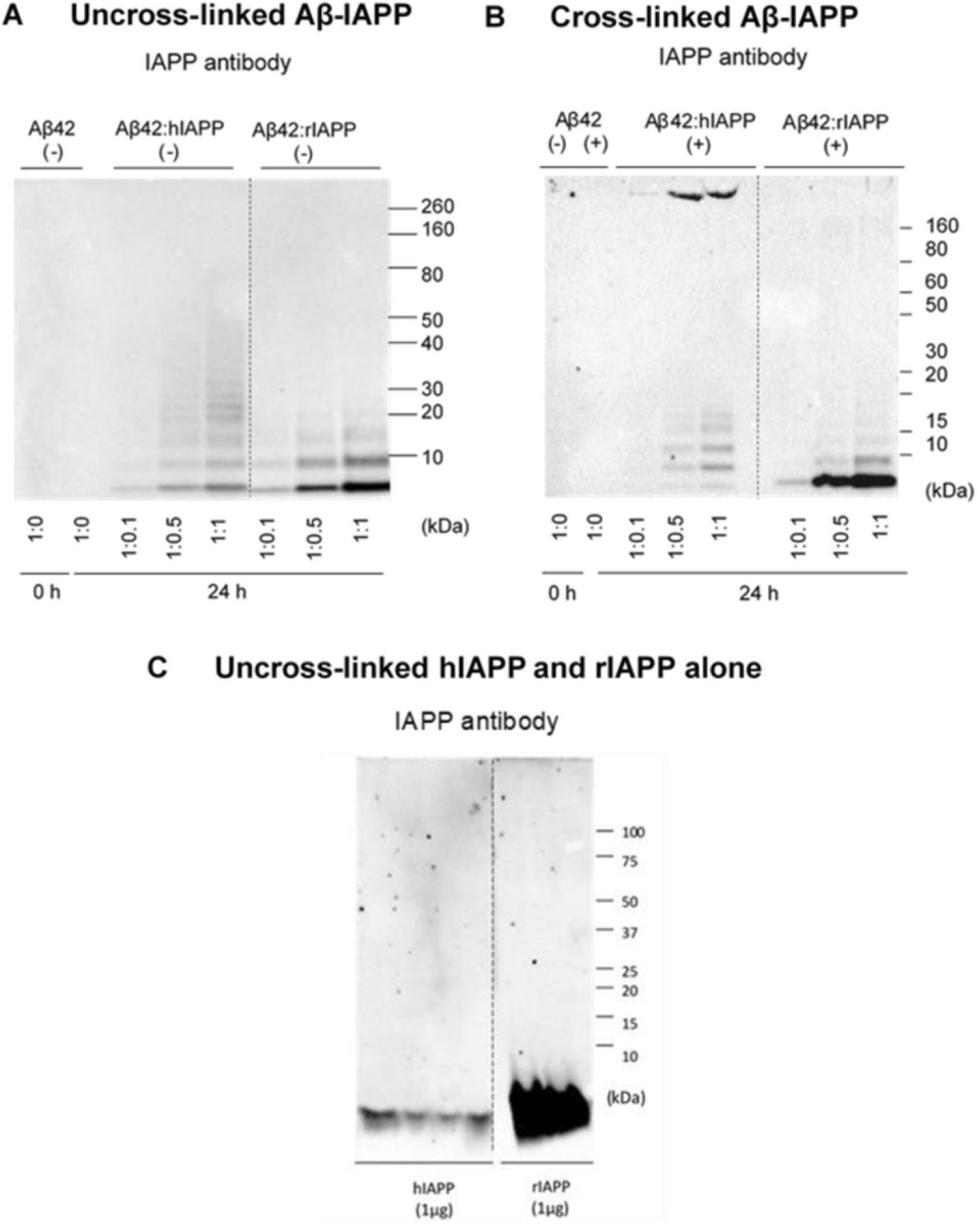
Size distribution of photo cross-linked Aβ42-IAPP heterocomplexes. Western immunoblotting analysis of (**A**) uncross-linked (−) and (**B**) cross-linked (+) Aβ42-IAPP combinations at 24 h timepoint assessed using an IAPP antibody (T-4157). (**C**) uncross-linked hIAPP and rIAPP assessed using IAPP antibody (T-4157). Aβ42 incubated with hIAPP at ratios 1:0.5 and 1:1 showed prominent small oligomers (dimer, trimer and tetramer) and large aggregates (>250 kDa), which were otherwise absent in hIAPP alone (compare **A–C**). The IAPP antibody detected formation of oligomers in Aβ-rIAPP mixtures, which were absent in rIAPP only, suggesting Aβ42 and rIAPP may be interacting to form heterocomplexes as well. Dotted lines indicate spliced bands from the same gel in which blank lanes were removed.

Findings above demonstrate that IAPP peptides alter Aβ42 aggregation and promote formation of distinct aggregates, possibly heterocomplexes of Aβ42-IAPP. To assess the heterocomplex formation, co-oligomerized Aβ42-IAPP samples were analysed using an IAPP antibody (T-4157) to determine whether protein bands detected by the Aβ antibody were cross-reactive to the IAPP antibody. Both CL and non-CL samples were analysed using the IAPP antibody. As expected, no signal was detected in the Aβ42 only lane and a very low signal was seen in Aβ42 incubated with hIAPP at 1:0.1 ratio (Fig. 3A). A more prominent signal was observed for Aβ42 incubated with hIAPP at ratios 1:0.5 and 1:1 showing small oligomers (dimer, trimer and tetramer) and large aggregates (>250 kDa), which were otherwise absent in hIAPP alone (compare Fig. 3A–C). Additionally, the IAPP antibody detected formation of oligomers in Aβ42-rIAPP mixtures, which were absent in rIAPP only (Fig. 3C)^31^. These findings demonstrated that both Aβ42-hIAPP and Aβ42-rIAPP mixtures contain distinct aggregate species that are immunoreactive to Aβ and IAPP specific antibodies, indicating the formation of heterocomplexes.

**Figure 4:**
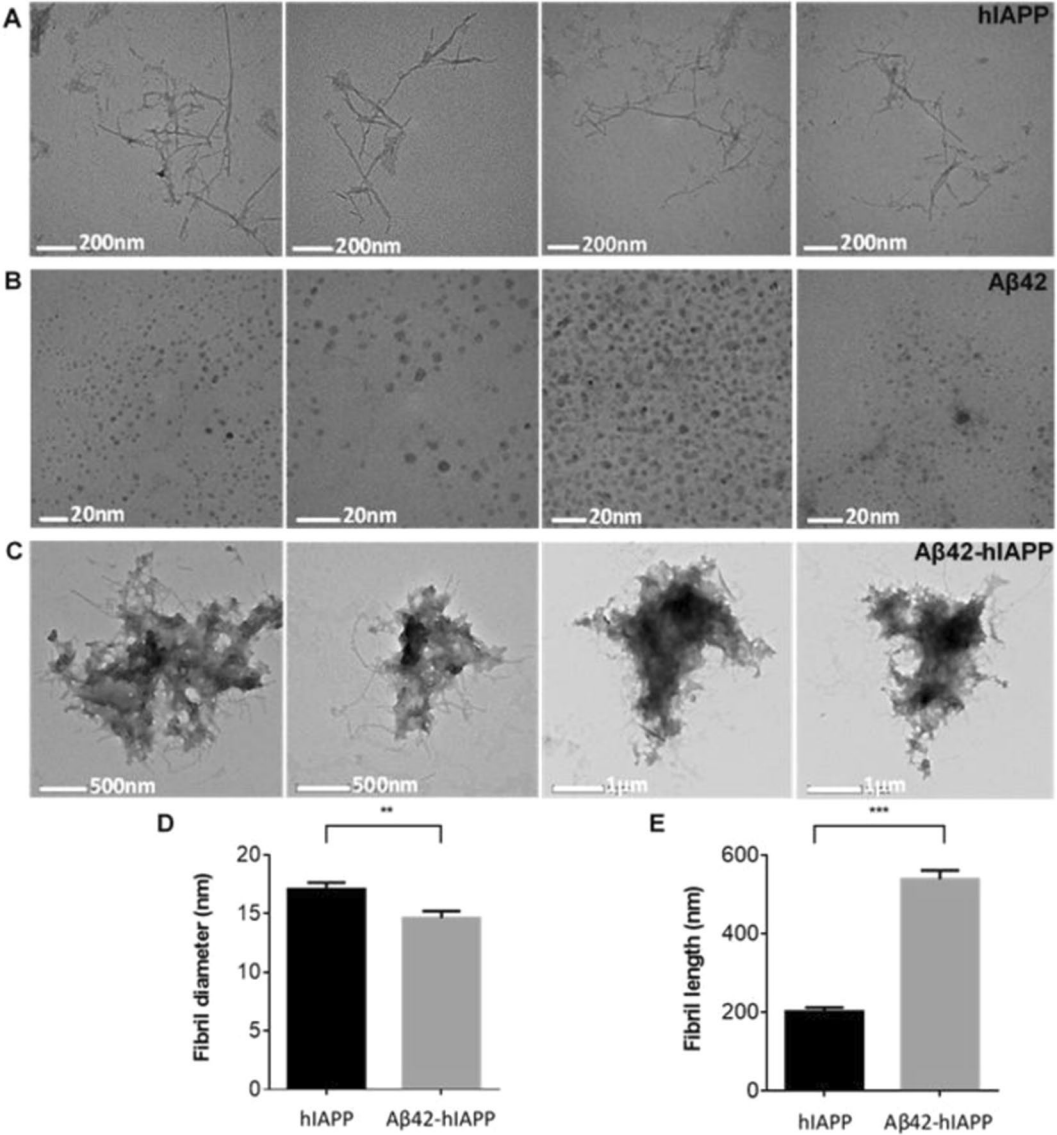
Electron micrographs of hIAPP, Aβ42 and Aβ42-hIAPP morphology. Electron micrographs demonstrating (**A**) fibril-like hIAPP (17 ± 0.5 nm in width, 202 ± 9.9 nm in length, n = 77) and spherical (**B**) Aβ42 oligomers (6 ± 0.2 nm in diameter, n = 207). (**C**) Aβ42-hIAPP formed large amorphous aggregates with distinct morphologies. Analysis of fibril (**D**) length and (**E**) diameter demonstrated that Aβ42-hIAPP (14 ± 0.6 nm in diameter, 539 ± 22.7 nm in length, n = 100) was significantly different from hIAPP (mean ± SEM, **p < 0.005, ***p < 0.001). Scale bar (**A**) 200 nm, (**B**) 20 nm, (**C**) 500 nm/1 µm. Aβ42-hIAPP mixtures are large amorphous structures that are distinctly different from either spherical Aβ42 oligomers or fibril-like hIAPP.

Aβ42, hIAPP and co-oligomerized Aβ42-hIAPP (1:1) mixtures were assessed by transmission electron microscopy (TEM). TEM analysis confirmed that hIAPP was mostly fibrillar (17 ± 0.5 nm in width, 202 ± 9.9 nm in length, n = 77) (Fig. 4A), while Aβ42 was predominately spherical oligomers (6 ± 0.2 nm in diameter, n = 207) (Fig. 4B). Aβ42-hIAPP mixtures were largely amorphous structures with extensions of thin fibre-like components surrounding the edges (14 ± 0.6 nm in width, 539 ± 22.7 nm in length, n = 100) (Fig. 4C). Notably, the fibres in Aβ42-hIAPP were significantly longer and thinner compared to those in hIAPP (p < 0.001) demonstrating that co-oligomerized Aβ42-hIAPP mixtures are morphologically distinct compared to hIAPP or Aβ42 isoforms alone (Fig. 4D,E). rIAPP and Aβ42-rIAPP (Fig. S1) mixtures were not included for TEM analysis in the present study as we were not able to observe any distinct structures. Overall, these findings demonstrate that Aβ42 and hIAPP interact to form heterocomplexes with distinct structural and morphological characteristics.

**Figure 5:**
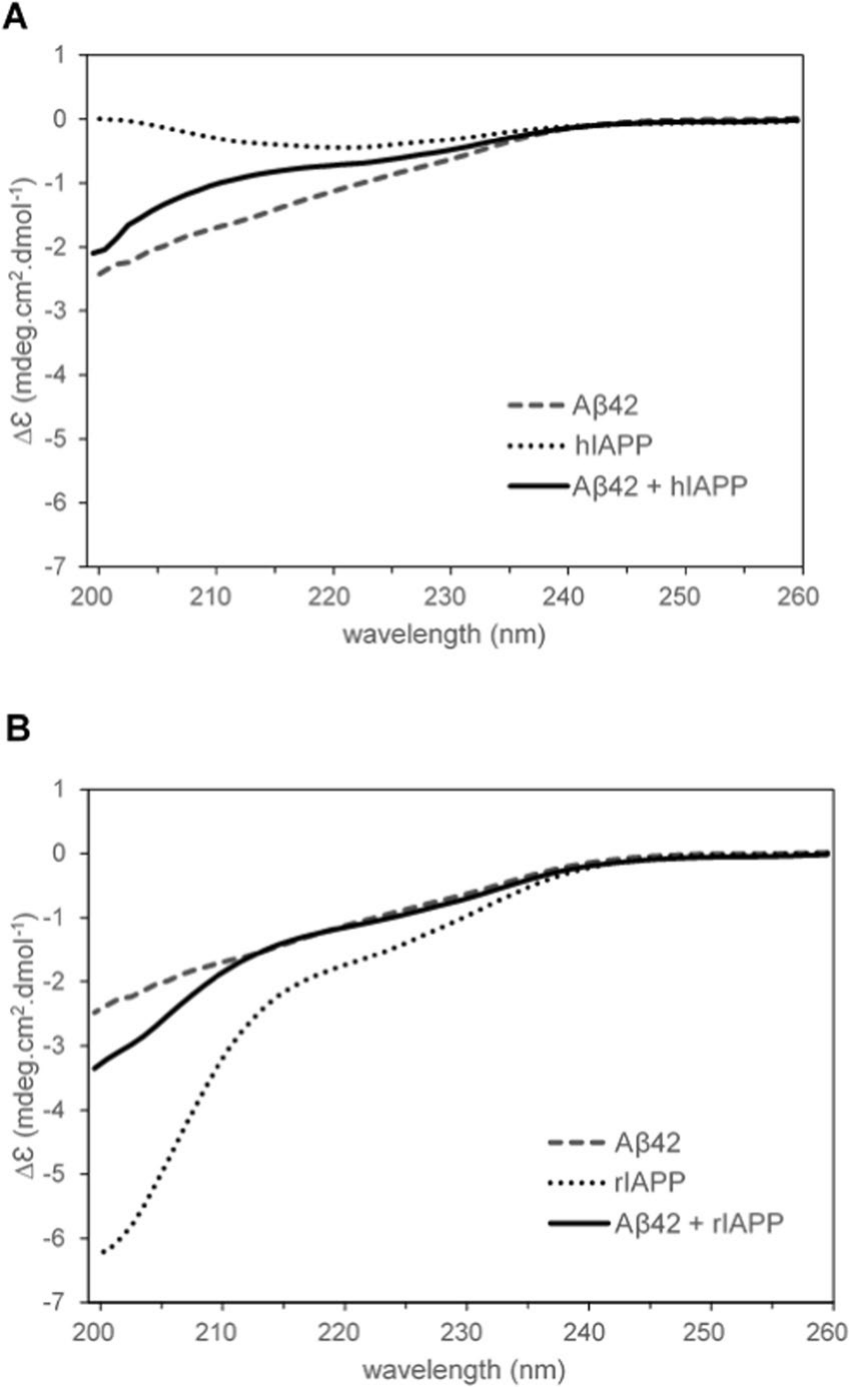
CD spectra Aβ42, IAPP and Aβ42-IAPP combinations between 200–260 nm. (**A**) CD spectra of Aβ42, hIAPP and Aβ42-hIAPP (1:1) mixtures. hIAPP had spectra characteristic for samples containing predominantly β-sheet, while Aβ42 and Aβ42-hIAPP contained predominantly random coil. (**B**) CD spectra obtained for Aβ42, rIAPP and Aβ42-rIAPP (1:1) mixtures demonstrated predominantly random coil. Each spectrum represents three biological replicates and nine technical replicates.

In addition, the secondary structure of the Aβ42-hIAPP aggregates were investigated using circular dichroism. Circular dichroism (CD) spectra were collected between 200 and 260 nm (Fig. 5) and the secondary structure of Aβ42, hIAPP, rIAPP, and their co-oligomerized mixtures estimated by JFIT CD analysis program (Table 1). Aβ42 has been predicted to contain 12% α-helix, 27% β-sheet, 61% random coil, which is consistent with the CD spectra shape and the NMR structure of the peptide^32^. Consistent with this, hIAPP was found to have a spectrum consistent with predominantly β-sheet. Conversely, the co-oligomerized Aβ42-hIAPP mixture had CD spectra characteristic for random coil (Fig. 5A). The CD data demonstrated that, unlike the amyloids formed by hIAPP, the aggregates of Aβ42 and hIAPP were amorphous. Furthermore, we measured CD spectra of rIAPP alone and co-oligomerized Aβ42-rIAPP mixtures. Unlike hIAPP, rIAPP displayed spectra consistent with predominant random coil. These results are consistent with the NMR structure of rIAPP^33^ and the peptide remaining soluble during incubation. The co-oligomerized Aβ42-rIAPP mixture was observed to have spectra consistent with predominantly random coil (Fig. 5B). Similar to Aβ42-hIAPP, CD spectra indicated that Aβ42-rIAPP mixtures were amorphous. However, the Aβ42-hIAPP mixture displayed more β-sheet character than the Aβ42-rIAPP mixture, reflecting the high β-sheet content of hIAPP. Overall, the addition of Aβ42 to hIAPP results in a reduction in β-sheet character as compared to hIAPP alone. While, Aβ42 addition to rIAPP maintains the predominance of coiled-coil character of rIAPP alone.

**Table 1:** Secondary structure of Aβ42, hIAPP, rIAPP, and their co-oligomerized mixtures estimated by JFIT CD analysis program.

Sample	% α-helical	% β-sheet	% random coil	R-value (%)
Aβ42	12.12 ± 2.03	27.25 ± 2.39	60.64 ± 2.73	12.2
hIAPP	10.36 ± 2.03	60.17 ± 7.84	29.47 ± 5.94	39.3
Aβ42 and hIAPP, 1:1	10.84 ± 1.14	25.69 ± 1.21	63.47 ± 1.58	11.7
rIAPP	19.75 ± 0.38	10.11 ± 0.80	70.14 ± 0.55	16.0
Aβ42 and rIAPP, 1:1	15.33 ± 1.53	20.38 ± 2.10	64.29 ± 1.00	13.3

The error bars show +/- SEM; each value represents three biological replicates and nine technical replicates.

**Figure 6:**
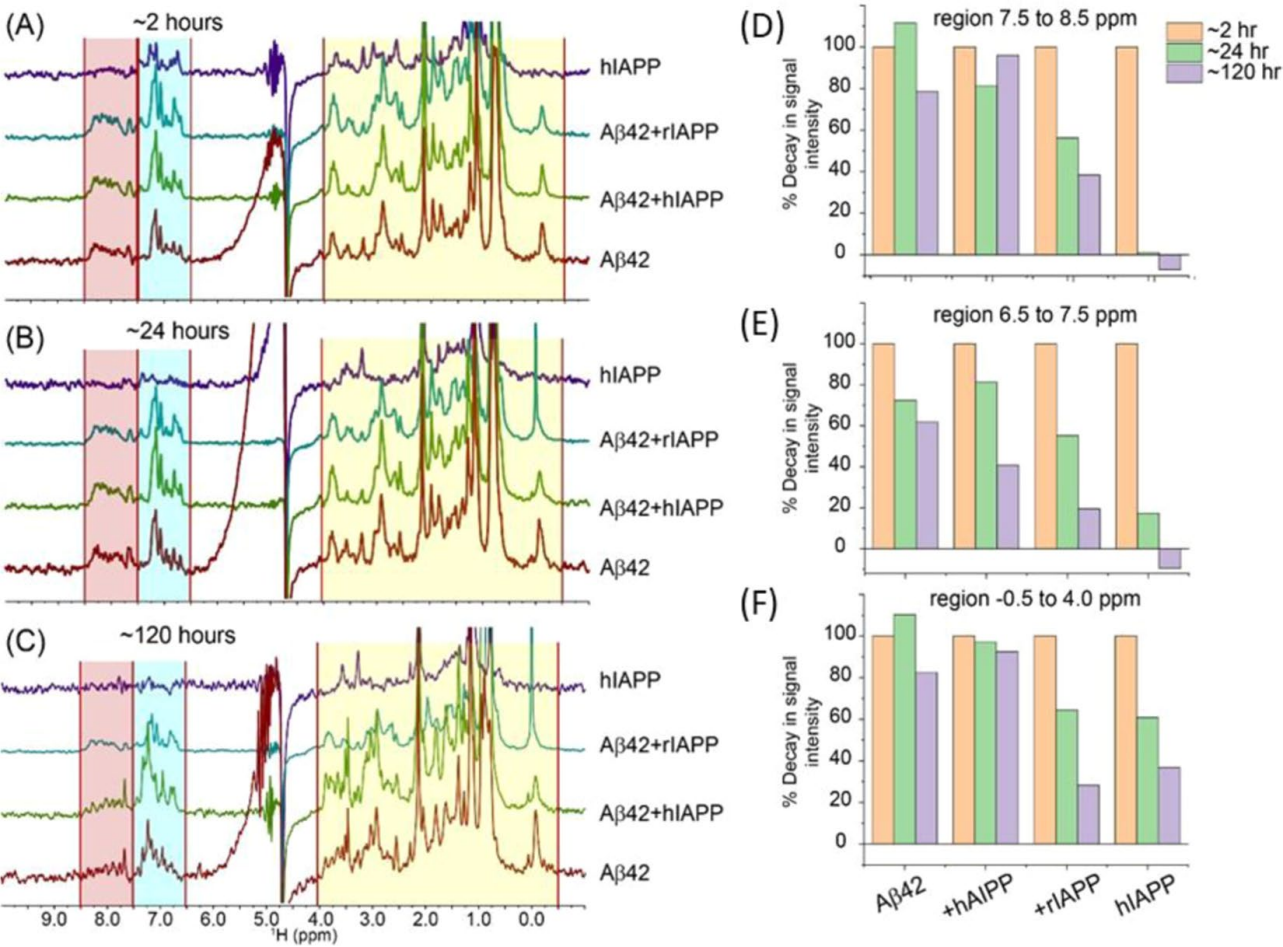
Monitoring the aggregation kinetics of Aβ42-IAPP combinations by ^1^H NMR. (**A**–**C**) Time-lapse ^1^H NMR spectra of 25 µM Aβ42 co-incubated with 25 µM hIAPP or rIAPP. NMR samples were prepared using 10 mM NaPi buffer, pH = 7.4 containing 10% D_2_O and spectra were recorded on a 500 MHz spectrometer at 25 °C. (**D**–**F**) Decay of ^1^H NMR signal intensities calculated from the spectra shown in (**A**–**C**) as a function of time as indicated by colors. The integrated total signal intensities of the selected regions highlighted in (**A**–**C**) were analyzed using MestReNova.

To provide further evidence for the interaction between Aβ42 and IAPP and the formation of heterocomplex aggregates, aggregation kinetics of Aβ42 was monitored in the presence of hIAPP or rIAPP using 1D proton NMR experiments. A freshly dissolved 25 µM Aβ42 or hIAPP exhibited a distinct spectral resolution in the ≈ 6.5 to 8.5 ppm (H^N^ + aromatic) region during the initial 2 h (Fig. 6A). Specifically, Aβ42 showed several well-resolved amide-proton peaks in the ≈ 7.5 to 8.5 ppm region (Fig. 6A, red). In contrast, very few peaks were observed for hIAPP at this time-point (Fig. 6A, purple). This is in agreement with reported results for the fast aggregation kinetics of hIAPP at pH 7.4 due to a reduced positive charge (~ + 1.7) as compared to that observed at pH 5.5 (~ + 3.0)^34^. An equimolar mixture of Aβ42 and hIAPP (green) or rIAPP (cyan) resulted in the appearance of peaks in the ≈ 6.5 to 8.5 ppm region at 2 h. This observation suggests either Aβ42 + hIAPP forms low-aggregate complexes that are in the detectable range of solution NMR spectroscopy, or the well-resolved peaks primarily consist of Aβ42 (Fig. 6A, green and cyan). In contrast, after 24 and 120 h of incubation, hIAPP showed no observable peaks whereas the Aβ42 + hIAPP mixture showed broad but detectable peaks. On the contrary, Aβ42 + rIAPP mixture presented a well-resolved ^1^H spectrum after 24 and 120 h of incubation (Fig. 6B,C). Considering the aggregating and non-aggregating propensities of hIAPP and rIAPP, respectively, the observed peaks are most likely from the relatively less aggregating species i.e. rIAPP (in Aβ42 + rIAPP) and Aβ42 (in Aβ42 + hIAPP).

The above notion was next tested by integrating the total intensity of the ^1^H peaks observed in the H^N^ + aromatic region. After 24 h incubation, Aβ42 showed several distinct trends in the intensity plot that include a slight increase in aliphatic and amide proton intensities followed by a decrease (~ 20–40% in the H^N^ + aromatic region) in intensity at 120 h (Fig. 6D–F). In contrast, hIAPP was associated with a significant reduction in the total intensity indicating its aggregation at pH 7.4. Whereas, the Aβ42 + rIAPP sample mixture presented a ~ 50% reduction (at 24 h) in signal intensity for the H^N^ + aromatic protons (Fig. 6D–F). This indicated that the observable peaks in Aβ42 + rIAPP are most likely from rIAPP that is known to be non-amyloidogenic. Interestingly, intensity plot for Aβ42 + hIAPP showed a relatively small change (~ 10% decay) in amide proton intensity after 120 h incubation as compared to Aβ42 or hIAPP alone in solution (Fig. 6D). This ruled out the assumption that observable peaks in the Aβ42 + hIAPP mixture are from Aβ42, indicating the formation of stable Aβ42-hIAPP heterocomplexes in solution. In fact, a ~ 60% decay in aromatic proton intensity indicated a possible involvement of aromatic side-chains in the complex formation (Fig. 6E).

**Figure 7:**
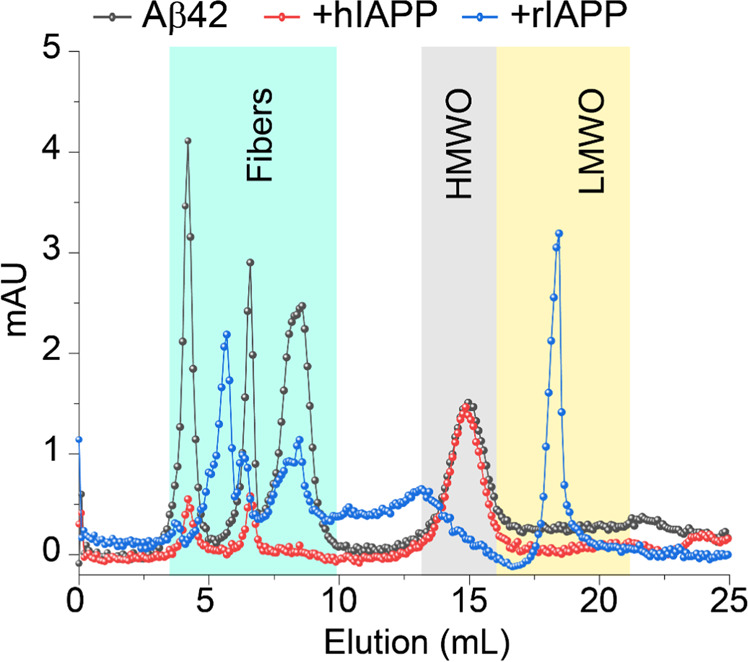
Size profiling of Aβ42-IAPP combinations using SEC. Size-profile analysis using SEC of 50 µM Aβ42 co-incubated with 50 µM hIAPP or rIAPP as indicated in colors. The SEC samples were prepared using 10 mM NaPi buffer, pH = 7.4 and incubated for ~ 24 h under continuous agitation prior injection onto the column. The HMWO and LMWO refer to the high-molecular and low-molecular weight oligomers, respectively.

Next, size-exclusion chromatography (SEC) was carried out to characterize the aggregated products of Aβ42 in the presence or absence of hIAPP or rIAPP (Fig. 7). At 50 µM Aβ42, a mixture of high-molecular weight oligomers (HMWO) and fibers were eluted at ≈ 13–17 and ≈ 3–10 mL, respectively (grey curve). 50 µM Aβ42 individually and mixed with equimolar of hIAPP showed no peaks in the region ≈ 18–20 mL indicating the absence of monomers or low-molecular weight oligomers (LMWO). Importantly, as compared to Aβ42 alone, the Aβ42 + hIAPP peptide solution was associated with low A_280_ absorbance in the region ≈ 3–10 mL indicating the presence of small amounts of fibers or large aggregates, but yielded an equivalent amount of HMWO as observed for Aβ42 alone (Fig. 7, red curve). Notably, 50 µM Aβ42 mixed with equimolar of rIAPP presented a sharp peak near ≈ 18–20 mL which is most likely from the non-aggregated rIAPP monomers or LMWO (Fig. 7, blue curve). In addition, the SEC profile represented the presence of Aβ42 aggregated fibers and oligomers that have a relatively low A280 absorbance in Aβ42 + rIAPP solution.

### Co-oligomerized Aβ-hIAPP mixtures promote cell death in SH-SY5Y neuroblastoma cells

**Figure 8:**
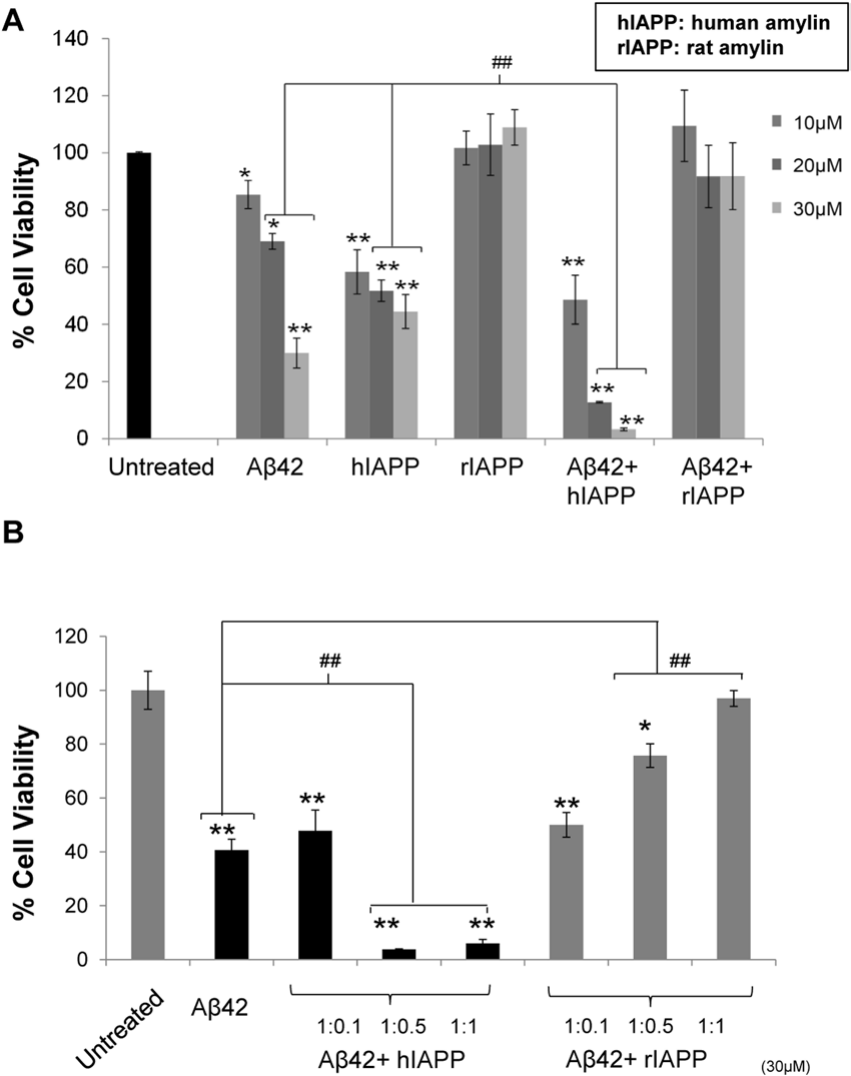
Toxicity of co-oligomerized Aβ42-IAPP mixtures in neuronal cells. Toxicity of Aβ42 and IAPP peptide mixtures in human neuroblastomas (SH-SY5Y) was assessed by MTS (soluble tetrazolium) assay. Cells were treated with (**A**) increasing concentrations (10, 20 and 30 µM) of Aβ42, hIAPP and Aβ42 + hIAPP (1:1) mixtures or (**B**) Aβ42 co-oligomerized with increasing concentration ratios of IAPP peptides (1:0.1, 1:0.5 and 1:1). Aβ42, hIAPP and Aβ42 + hIAPP treatment showed dose dependent cell death compared to rIAPP and Aβ42 + rIAPP, as indicated by the reduced ability of neurons to metabolize MTS (*p < 0.05, **p < 0.01). At higher concentrations (20 and 30 µM), Aβ42 + hIAPP showed significantly higher cell death as compared to Aβ42 and hIAPP only (^##^p < 0.01). In contrast to hIAPP, rIAPP rescued cells from Aβ42 induced toxicity. No significant loss of cell viability was observed with different concentrations of Aβ42-rIAPP treatment. At higher concentration ratios of hIAPP (1:0.5 and 1:1), Aβ42-hIAPP mixtures showed a 3–4 fold increase, whereas rIAPP showed a dose dependent decrease in cell death with increase concentrations of rIAPP in Aβ42-rIAPP mixtures.

We have shown above that the combination of Aβ42 and hIAPP promotes the formation of heterocomplex aggregates with a distinct morphology from that seen in Aβ42 or hIAPP only preparations. To determine if these heterocomplex aggregates display altered neurotoxicity compared to Aβ42 and hIAPP alone, SH-SY5Y cells were treated with Aβ42, hIAPP, rIAPP, Aβ42 + hIAPP or Aβ42 + rIAPP preparations and cell death was evaluated using MTS assay^35,36^. We first assessed the dose response of Aβ42, hIAPP, rIAPP and co-oligomerized Aβ42 + hIAPP/rIAPP (1:1 ratio) (Fig. 8A). To compare the toxicity of the individual peptides with the co-oligomerized mixtures, the total amount of peptide was maintained the same in the individual and combination peptide treatments. For the individual treatments (Aβ42, hIAPP, rIAPP), increasing doses of the peptides was used (10, 20 and 30 µM). For the combination peptide treatments (Aβ42 + hIAPP/rIAPP), equivalent doses by weight was used, but with 1:1 ratio of each peptide. A dose dependent reduction in cell viability was observed for Aβ42, hIAPP and Aβ42 + hIAPP solutions whereas rIAPP and Aβ42 + rIAPP showed similar viability to vehicle treated cells (Fig. 8A). Notably, a marked 3-fold reduction in cell viability was observed for cells treated with 20 or 30 µM of Aβ42 + hIAPP combination compared to those treated with Aβ42 or hIAPP only. Cells treated with Aβ42-rIAPP complexes (10–30 µM) showed no significant loss in viability compared to the control, indicating a protective role for rIAPP in reducing Aβ42 induced cell death.

Above we show that increasing doses of the Aβ42: hIAPP combination promoted cell death, whist Aβ42: rIAPP protected against cell death. To determine whether hIAPP and rIAPP modulated this effect in a dose dependent manner, Aβ42 was co-oligomerized with increasing doses of hIAPP or rIAPP peptides, prior to treating cells (Fig. 8B). Cell viability after treatment with Aβ42:hIAPP (1:0.1 ratio) was similar to cells treated with Aβ42 alone. However, a 3 fold increase in cell death was observed in cells treated with Aβ42:hIAPP combinations at ratios 1:0.5 and 1:1. In contrast, viability of cells treated with Aβ42-rIAPP mixtures, increased with higher concentrations of rIAPP in the co-oligomerized mixtures (Fig. 8B). Overall, these data were consistent with findings from CD, NMR and SEC analysis and corroborated with our peptide aggregation studies, showing that hIAPP enhances, but rIAPP reduces Aβ aggregation and associated neurotoxicity.

**Figure 9:**
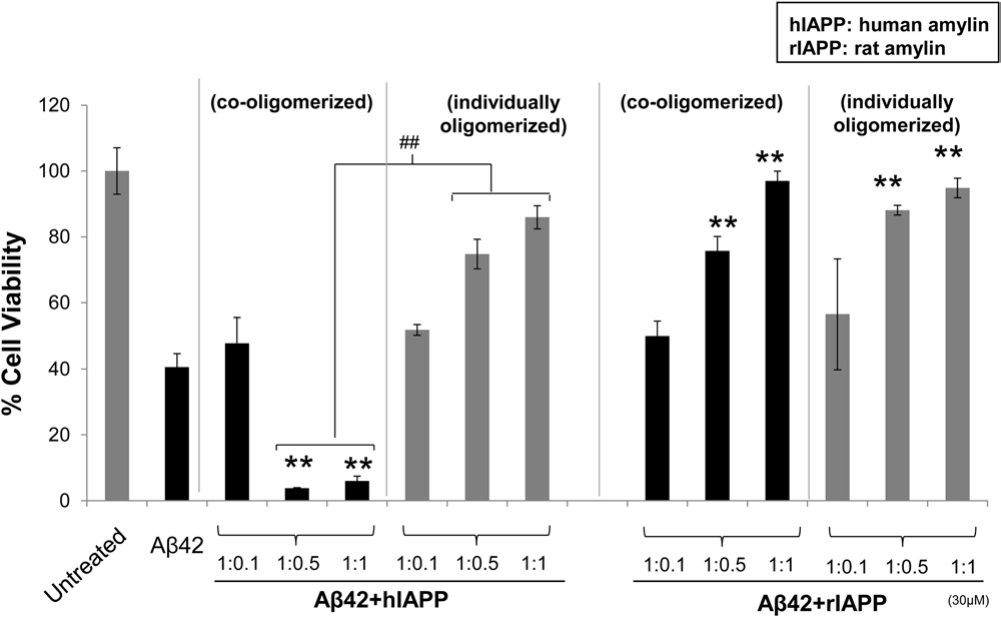
Toxicity of co-oligomerized and individually oligomerized Aβ42-IAPP mixtures. Toxicity of co-oligomerized and independently oligomerized Aβ42 and IAPP peptides were assessed in human neuroblastomas (SH-SY5Y) was assessed by MTS (soluble tetrazolium) assay. Co-oligomerized Aβ42-hIAPP showed 3–4 fold increase in cell death as compared to Aβ42 only (^**^p < 0.01). However, when individually oligomerized, Aβ42-hIAPP treatments showed less cell death as compared to Aβ42 only (^##^pp < 0.01). Notably, rIAPP rescued Aβ42 induced toxicity in a dose-dependent manner in both pre-incubated and non-pre-incubated conditions tested (**p < 0.01).

To further investigate whether increased toxicity of Aβ42 + hIAPP mixtures is dependent on their ability to interact and form heterocomplex aggregates, we tested cell viability of SHSY-5Y cells treated with co-oligomerized and individually oligomerized Aβ42 and IAPP peptides (Fig. 9). For preparing co-oligomerized samples, freshly prepared Aβ42 and IAPP peptides were co-incubated for 24 h at 4 °C to interact and form heterocomplexes, followed by cell treatment. For preparing individually oligomerized peptides, freshly prepared Aβ42 and IAPP peptides were incubated separately for 24 h at 4 °C and mixed only prior to cell treatment. As observed in Fig. 8, co-oligomerized Aβ42-hIAPP showed 3 fold increased cell death as compared to Aβ42, whereas Aβ42-rIAPP showed significantly less cell death. Notably, rIAPP reduced Aβ42 induced cell death in a dose-dependent manner in both co-oligomerized and individually oligomerized conditions tested. Interestingly, rIAPP and also hIAPP showed a reduction in oligomer Aβ42 induced neuronal cell death, when the peptides were individually oligomerized prior to treatment (Fig. 9). These findings suggested that Aβ42-hIAPP co-oligomerization is essential for its enhanced toxicity, whereas Aβ42-rIAPP combination showed significantly reduced toxicity irrespective of whether it was co-oligomerized with Aβ42 or when both peptides were individually oligomerized.

## Discussion

The interactions of the intrinsically disordered proteins islet amyloid polypeptide (hIAPP) with beta amyloid (Aβ) peptide are crucial to understanding the pathogeneses of AD and T2D. hIAPP can act as a seed for Aβ aggregation and studies predict that Aβ and hIAPP interact to form various heterocomplex structures. But whether these heterocomplexes have altered structure, size distribution and toxicity are largely unknown. Here we demonstrate a dose-dependent effect of hIAPP in increasing Aβ aggregation and formation of small and high molecular weight (HMW) aggregates. Oligomerization and photo-crosslinking analysis of Aβ-IAPP mixtures reveal the formation of small oligomer heterocomplexes ranging from 10–40 kDa and large aggregate heterocomplexes greater than 250 kDa. TEM analysis confirmed the formation of large amorphous aggregates in Aβ42-hIAPP mixtures that were morphologically distinct from the spherical Aβ oligomers and fibrillar hIAPP, providing further evidence for Aβ42-hIAPP heterocomplex formation. In addition, CD analysis confirmed the formation of Aβ42-hIAPP heterocomplexes with secondary structure distinct from Aβ oligomers and fibrillar hIAPP. Notably, the CD spectra of the co-oligomerized Aβ42:hIAPP was not the average of the spectra of the two peptides, demonstrating a functional interaction between Aβ42 and hIAPP to form a distinct structure. Analysis of Aβ42-hIAPP mixtures by ^1^H NMR experiments indicated a possible involvement of aromatic side-chains in the formation of these heterocomplex aggregates. Overall, our findings demonstrate the formation of two main isoforms of Aβ42-hIAPP heterocomplexes, including small oligomers possibly formed by strong monomer-monomer interaction and the large amorphous aggregates formed by weaker oligomer-oligomer or oligomer-monomer interaction^21,22,24,25^.

Both Aβ42 and hIAPP are neurotoxic but our findings demonstrate that co-oligomerized Aβ42-hIAPP increased neuronal cell death by up to 3-fold, as compared to Aβ42 or hIAPP alone. In addition, we show that co-oligomerization of Aβ42-hIAPP is essential for its increased ability to induce neuronal cell death. It is notable that Aβ42-hIAPP heterocomplex mixtures showed increased cell death, despite formation of large amorphous aggregates, which are generally considered to be less toxic compared to the soluble oligomers. One possible mechanism underlying the increased toxicity of the Aβ42-hIAPP heterocomplexes is their enhanced ability to interact with neuronal cell membranes and cause damage. In fact, we recently reported a dose-dependent effect of hIAPP in promoting the uptake of Aβ42 in neuronal cells (SHSY-5Y), indicating that Aβ42-hIAPP heterocomplexes have an enhanced ability to interact and permeabilize cell membranes^37^. A recent report also demonstrated that hIAPP-Aβ heterocomplexes adsorb, aggregate, and permeabilize the isolated β-cell membrane significantly slower than pure hIAPP, however, at a rate that is much faster than that of pure Aβ^38^. Analysis of interaction of hIAPP, Aβ and a mixture of both with an anionic model raft membrane showed a dominant effect of hIAPP on the aggregation process and on the hydrogen-bonding pattern of the assemblies present in the mixture. The analysis of the interaction of Aβ with a non-amyloidogenic IAPP mimic confirmed these findings^38,39^. In addition, recent molecular dynamics simulations (MD) studies indicates that Aβ-hIAPP interacts more strongly with lipid bilayers facilitated by electrostatic interactions and formation of Ca^2+^ bridges^40^. These findings suggest membrane damage and cell permeabilization as important mechanisms for Aβ42-hIAPP heterocomplex-mediated toxicity.

Another potential mechanism underlying the increased toxicity of Aβ42-hIAPP heterocomplexes in neuronal cells is their ability to bind certain cell receptors or modulate signalling pathways that may exacerbate cell dysfunction and death. Recent evidence indicates that Aβ is a specific agonist of the AMY3 receptor (amylin receptor 3) and can mediate toxicity via this receptor^41^. AMY receptor antagonists AC253, AC187 (truncated IAPP peptides lacking the functional amyloidogenic core sequences) have been shown to block Aβ induced toxicity^42–44^. AMY receptors are putative targets for IAPP peptides and are composed of calcitonin receptor (CTR) and one of the three receptor activity-modifying proteins (RAMP1–3), resulting in receptor subtypes AMY1–3^45^. Both IAPP and Aβ can allosterically activate AMY receptors and both the peptides may be present in monomeric, oligomeric, or fibrillar forms which can differentially activate/modulate AMY receptor subtypes. Our findings therefore raise the possibility that certain Aβ42-hIAPP heterocomplex structures may have increased ability to bind AMY receptor subtypes and promote cell death pathways. However, further investigation is required to determine the interacting domains of Aβ and hIAPP with AMY3 receptor and whether Aβ42-hIAPP complexes have increased binding affinity and ability to modulate AMY3 receptor activity and other subtypes. Overall, these studies in combination with our findings demonstrate that in addition to promoting Aβ aggregation in the brain, hIAPP can form highly toxic heterocomplexes with Aβ42 that have the potential to exacerbate neurodegeneration in the brain.

Despite its pathogenic role in promoting Aβ accumulation and toxicity, recent reports provide evidence for the benefits of IAPP in AD mouse models^46,47^. Peripheral treatment with IAPP lowered the concentrations of Aβ in the brain^46^, reduced multiple pathological markers and improved learning and memory in AD mouse model^47^. It is also suggested that the protective effects of IAPP require interaction with its cognate AMY receptor. Our data demonstrates a reduction in oligomer Aβ mediated neuronal cell death by both hIAPP and rIAPP, independent of their ability to modulate Aβ aggregation. Although non-aggregating IAPP-based peptides such as pramlintide, has received significant interest in developing treatment strategies for AD^3,31,48–50^, they can have a tendency to form fibrils^48^ and also cross-seed with Aβ that may have undesirable effects^31^. Although rIAPP reduces toxicity, we demonstrate in our cross-linking experiments that it may also interact with Aβ to form stable oligomer heterocomplexes, similar to Aβ42-hIAPP. Formation of heterocomplexes can impact the clearance processes and the normal physiology of Aβ in the brain raising concerns regarding the use of IAPP mimetics in AD therapeutic strategies. A detailed investigation of the IAPP peptide domains that are critical for self-aggregation, interaction with Aβ, AMY receptor subtypes and their ability to reduce oligomer mediated cell death, will therefore be important for designing improved mimetics and IAPP based treatment strategies for the management of AD and T2D.

## Materials and Methods

### Peptide preparation

Lyophilised synthetic peptides (Aβ1-42, hIAPP and the negative control rIAPP) were purchased from the ERI Amyloid Laboratory, LLC (Oxford, CT, USA). Peptides were prepared according to established protocols^51^. Briefly, dry peptides were weighed and dissolved in 1,1,1,3,3,3-hexafluoro-2-propanol (HFIP; Sigma-Aldrich, USA) for 1 h at room temperature (RT) and then separated into 450 μg aliquots. The HFIP was left to evaporate overnight at RT, producing dry peptide films which were stored at –20 °C for future use. Before each experiment, fresh Aβ and IAPP solutions were prepared by initially solubilising the dry peptide in 20 μl dimethyl sulfoxide (DMSO; Merck Millipore, USA) to make a 5 mM stock. Resolubilised peptides were centrifuged at 14 000 g for 1 min at RT (sealed with parafilm to prevent water infiltration) and then bath sonicated using a Bioruptor® Plus sonication device (Diagenode Incorporated, USA) for 10 min at 18 °C (40 cycles; 10 secs on and 5 secs off). This was followed by addition of 980 μl ice-cold Ham’s F12 phenol red-free media (Atlanta Biologicals, USA) to make a final concentration of 100 μM. The peptide solution was incubated at 4 °C for 24 h to allow formation of oligomers.

### Kinetic thioflavin-T assay

Peptides were prepared according to previously established protocols^35^, with minor modifications (addition of 980 μl ice-cold TBS filtered through a 0.22 µM syringe filter). Fluorescence was measured using a FLUOstar Omega Plate Reader (BMG Labtech, Ortenberg, Germany) with excitation and emission maxima set at 450 nm and 490 nm, respectively. Aβ42 was prepared in combination with increasing concentration ratios (1:0, 1:0.1, 1:0.5 or 1:1) of hIAPP or rIAPP in sterile black 96-well culture plates. 20 µl of Aβ-IAPP mixtures were incubated with 80 µl of 6 µM thioflavin-T (ThT) solution (Sigma-Aldrich, USA) for 26 h and fluorescence readings were recorded in 10 min intervals. The system was maintained at RT during the time course of the experiments and included circular agitation between measurements. Protocol also includes gain adjust to the highest well (hIAPP only). Intraexperimental groups were performed in quadruplicate and independent experiments were repeated three times.

### SDS-PAGE and western immunoblotting analysis

Aβ peptides were prepared with increasing concentrations of hIAPP and rIAPP (as above). Samples were collected at 0 h and 24 h time-points and were analysed by gel electrophoresis and western immunoblotting. Immunoblot analysis was conducted as per previously established protocols^36^.

### Photo-induced cross-linking of unmodified proteins

Photo-induced cross-linking (PICUP) was performed following established protocols with minor modifications^30^. Irradiation was accomplished using a Samsung S6 Camera (SM-G9201) and irradiation time consisted of 4 ×1 sec pulses at 5 cm distance. For the reaction, 2.5 µl of 1 mM Tris(2,2-bipyridyl) dichlororuthenium(II) hexahydrate (RuBpy; Sigma-Aldrich, USA) and 2.5 µl of 20 mM ammonium persulfate (APS) in buffer (10 mM sodium phosphate, pH 7.4) were added to 50 µl of peptide solution for a total volume of 55 μl. After the mixture was irradiated, the reaction was quenched immediately with 45 µl 2x NuPAGE^TM^ LDS sample buffer (ThermoFisher Scientific, USA) containing 5% β-mercaptoethanol (Sigma-Aldirch, USA). Uncross-linked peptides were exposed to the same conditions, without the addition of cross-linking reagents, for comparison. Photo-cross-linking was followed by size fractionation using SDS-PAGE and detection by immunoblotting analysis.

### Transmission electron microscopy

Peptides were prepared as detailed above. All solvents used were filtered through 0.22 µM syringe filters (Corning Incorporated, USA). Peptide solutions were prepared at a 1:1 ratio and diluted in deionised water for imaging analysis (Aβ at 1/3, Aβ-hIAPP at 1/4 and hIAPP at 1/5). Diluted samples (15 μl) were applied to carbon coated 400-mesh copper grids (2SPI^®^ Supplies, West Chester, Pennsylvania, USA) which had been glow discharged in nitrogen. Excess sample was removed with filter paper and grids were washed. A second layer of sample was applied (10 ul) and incubated for 10 min. After washing, grids were negatively stained with 10 ul of 2% aqueous uranyl acetate (ProSciTech, Queensland, Australia). Grids were examined using a FEI TALOS^TM^ FS200X G2 Field Emission Gun Transmission Electron Microscope at 200 kV. Micrographs were recorded using a Ceta 16 M CCD camera (FEI, USA). ImageJ Software Version 2006.02.01 (Broken Symmetry) was used to quantify and analyse micrographs.

### Circular dichroism

Peptides were prepared as previously outlined with minor modifications (dry peptides were solubilised in 20 μl NaOH [20 mM] with the addition of 980 μl PBS). Circular dichroism was measured at 20 °C on a CD spectropolarimeter (Jasco J-810) equipped with a Peltier heating environment for Aβ42, hIAPP, rIAPP, and their co-oligomerized mixtures (1:1). Data were recorded using a 1 mm quartz cuvette (Hellma) and 200–260 nm measurement range, 1 nm bandwidth, 3 s response time, and a 1 nm data pitch. Data were de-convoluted into secondary structure content using the JFIT analysis program.

### Nuclear magnetic resonance spectroscopy and size exclusion chromatography

The synthetic Aβ42, human and rat amylin peptide powder (≈ 1 mg/mL) were treated with HFIP and incubated for ≈ 30 minutes on ice (Aβ42 was incubated at RT). The peptide sample was prepared as described elsewhere^34,52^. Briefly, several aliquots of HFIP treated peptide (0.1 mg/mL) were subjected to 48 h of lyophilization. The lyophilized powders were resuspended in 10 mM sodium phosphate (NaPi), pH 7.4 following spectroscopic measurement of concentration using a NanoDrop All NMR samples were prepared in NaPi buffer containing 10% deuterated water (v/v). ^1^H NMR spectra were obtained using 512 scans for 25 µM Aβ42 in the presence or absence of equimolar human or rat-IAPP with a recycle delay of 2 s. NMR experiments were carried out on a 500 MHz Bruker spectrometer equipped with a triple-resonance (1H, 13 C and 19 F) probe at 25 °C. All NMR spectra were processed using TopSpin 4.0.6 by referencing to the water proton peak at ≈ 4.7 ppm. NMR spectra were then analyzed and plotted using MestReNova 12.0.4.

For size distribution profiling, 50 µM Aβ42 dissolved in 1 mL of NaPi buffer was incubated in the presence or absence of equimolar amylin (human or rat) for 24 h under gentle shaking at room temperature. The sample was next injected onto a Superdex 75 (10/300GL) column (GE Healthcare) and eluted at a flow rate of 1 mL/min. The size-exclusion chromatography profiles were analyzed using the Origin program.

### Peptide treatment and cell viability analysis in neuronal cells

Toxicity of Aβ42 and IAPP peptides were assessed in SH-SY5Y cells as described previously^35^. Cells were seeded in 96-well plates, with a seeding density of 20,000 cells per well and incubated for 48 h maintained in a 5% CO_2_ incubator. Prior to treating the cells, the culture media was exchanged with fresh treatment media containing DMEM-F12 phenol red and supplemented with 1% FBS, L-Glutamine and Penicillin and streptomycin. Subsequently, cells were incubated with Aβ42, hIAPP, rIAPP, Aβ42-hIAPP and Aβ42-rIAPP combinations (co-oligomerized and individually oligomerized) at different concentrations, and then analysed by MTS assays to assess the cell viability. MTS (%viability) assays were performed according to established protocol published by Promega (USA). The MTS assay is a colourimetric sensitive quantification of viable cells in proliferation and cytotoxicity assay.

## Supplementary information

Supplementary information.
