# Supplementary material for: Amylin and beta amyloid proteins interact to form amorphous heterocomplexes with enhanced toxicity in neuronal cells
Source: Sci Rep. 2020 Jun 25;10:10356. doi: 10.1038/s41598-020-66602-9 (PMC7316712; doi:10.1038/s41598-020-66602-9)
Supplement: Supplementary file 1 — Supplementary information. [file 41598_2020_66602_MOESM1_ESM.docx]

**Amylin and beta amyloid proteins interact to form amorphous heterocomplexes with enhanced toxicity in neuronal cells**

Prashant Bharadwaj^1,2*^, Tanya Solomon^1*^, Bikash R. Sahoo^3^, Katarzyna Ignasiak^4^, Scott Gaskin^1^, Joanne Rowles^1^, Giuseppe Verdile^1, 2^, Mark J. Howard^5, 6^, Charles S. Bond^4^, Ayyalusamy Ramamoorthy^3^, Ralph Martins^2,7^, Philip Newsholme^1^

^1^ School of Pharmacy and Biomedical Sciences, Curtin Health and Innovation Research Institute (CHIRI), Faculty of Health Sciences, Curtin University, Western Australia, Australia, 6107

^2^ Centre of Excellence for Alzheimer’s disease Research and Care, School of Medical and Health Sciences, Edith Cowan University, Western Australia, Australia, 6027

^3^ Biophysics and Department of Chemistry, Biomedical Engineering, Macromolecular Science and Engineering, University of Michigan, Ann Arbor, MI, 48109-1055, USA^.^

^4^ School of Molecular Sciences, The University of Western Australia, Western Australia, Australia, 6009

^5^ Centre for Microscopy, Characterisation and Analysis, The University of Western Australia, Western Australia, Australia, 6009

^6^ School of Chemistry, University of Leeds, Leeds LS2 9JT UK

^7^ School of Biomedical Science, Macquarie University, Sydney NSW, Australia

*These authors contributed equally to this work

Corresponding Author: Dr Prashant Bharadwaj, p.bharadwaj@ecu.edu.au

**Supplementary Figures**


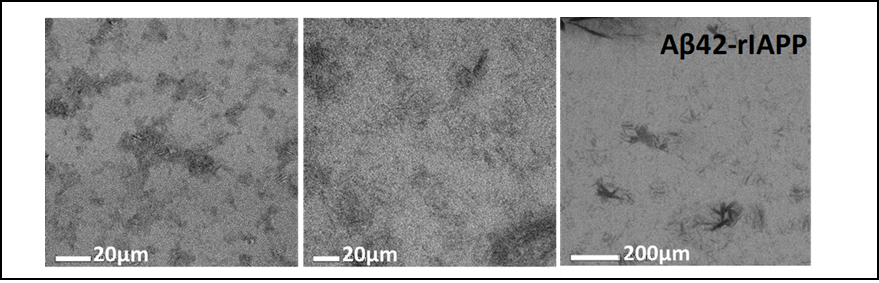


**Figure S1. Electron micrographs of Aβ42-rIAPP morphology.** Unlike Aβ42 and Aβ42-hIAPP, Aβ42-rIAPP mixtures did not display any distinct structures or morphology

**
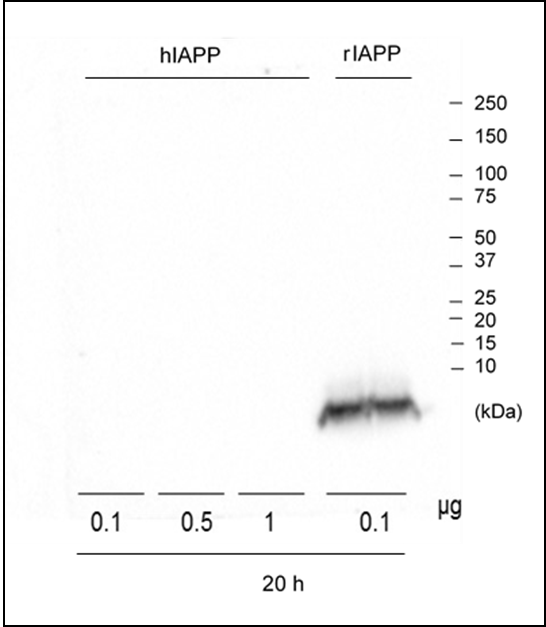
**

**Figure S2. Oligomerized hIAPP and rIAPP assessed using IAPP antibody (T-4157).** Western immunoblotting analysis of hIAPP (0.1, 0.5 and 1 μg) and rIAPP (1 μg) incubated for 20 h at 4°C. The rabbit anti-IAPP antibody (T-4157) demonstrated a higher binding affinity for rIAPP compared to hIAPP.
